# Role of the *Mycobacterium marinum* ESX-1 Secretion System in Sliding Motility and Biofilm Formation

**DOI:** 10.3389/fmicb.2018.01160

**Published:** 2018-05-30

**Authors:** Li-Yin Lai, Tzu-Lung Lin, Yi-Yin Chen, Pei-Fang Hsieh, Jin-Town Wang

**Affiliations:** ^1^Department of Microbiology, National Taiwan University College of Medicine, Taipei, Taiwan; ^2^Department of Pediatrics, Chang Gung Children’s Hospital, Chang Gung Memorial Hospital, College of Medicine, Chang Gung University, Taoyuan, Taiwan; ^3^Department of Internal Medicine, National Taiwan University Hospital, Taipei, Taiwan

**Keywords:** type VII secretion system, *Mycobacterium marinum*, ESX-1, sliding motility, biofilm formation

## Abstract

*Mycobacterium marinum* is a close relative of *Mycobacterium tuberculosis* that can cause systemic tuberculosis-like infections in ectotherms and skin infections in humans. Sliding motility correlates with biofilm formation and virulence in most bacteria. In this study, we used a sliding motility assay to screen 2,304 transposon mutants of *M. marinum* NTUH-M6885 and identified five transposon mutants with decreased sliding motility. Transposons that interrupted the type VII secretion system (T7SS) ESX-1-related genes, *espE* (*mmar_5439*), *espF* (*mmar_5440*), and *eccA1* (*mmar_5443*), were present in 3 mutants. We performed reverse-transcription polymerase chain reaction to verify genes from *mmar_5438* to *mmar_5450*, which were found to belong to a single transcriptional unit. Deletion mutants of *espE*, *espF*, *espG* (*mmar_5441*), and *espH* (*mmar_5442*) displayed significant attenuation regarding sliding motility and biofilm formation. *M. marinum* NTUH-M6885 possesses a functional ESX-1 secretion system. However, deletion of *espG* or *espH* resulted in slightly decreased secretion of EsxB (which is also known as CFP-10). Thus, the *M. marinum* ESX-1 secretion system mediates sliding motility and is crucial for biofilm formation. These data provide new insight into *M. marinum* biofilm formation.

## Introduction

*Mycobacterium marinum* is a non-tuberculous photochromogenic mycobacterium. It is a close relative of *Mycobacterium tuberculosis*, and also causes systemic tuberculosis-like infections in ectotherms (Solomon et al., 2003; Hagedorn and Soldati, 2007; Alibaud et al., 2011). Researchers first isolated *M. marinum* from saltwater fish in the 1920s and identified it as a human pathogen in the 1950s (Riera et al., 2015). This species usually occurs in warm saltwater, freshwater, and poikilothermic animals; fish, frogs, and amphibians are its main natural hosts. It grows best at a temperature of 25–35°C (McArdle et al., 2016). The prevalence of *M. marinum* infections in humans has risen in recent years because of the increasing popularity of home aquariums (Riera et al., 2015). *M. marinum* infections most commonly occur on the skin, especially the extremities, because of the low-temperature requirements of the bacteria for growth (Oh et al., 2015). An *M. marinum* skin infection is referred to as an aquarium granuloma, swimming pool granuloma, or fish tank granuloma (Solomon et al., 2003; Meijer, 2015; Sette et al., 2015).

*M. marinum* infection usually occurs following contact with an infected animal or handling of contaminated aquariums or water. It can also occur as an opportunistic infection, primarily in immune-deficient patients, such as those with human immunodeficiency virus (HIV)/acquired immunodeficiency syndrome (AIDS) (Ren et al., 2007; Rombouts et al., 2010). Clinicians characterize the stages of *M. marinum* infections as the initial stage (type I), in which there are single or multiple skin papules or nodules; advanced stage (type II), in which there are granulomas; and severe stage (type III), in which immunosuppressed patients experience tenosynovitis, arthritis, or osteomyelitis (Riera et al., 2015). The diagnosis of *M. marinum* infection is often delayed, due to its low prevalence or because there are few specific clinical signs and symptoms. Thus, many patients initially receive incorrect diagnoses and inappropriate treatments.

Many mycobacteria can spread on a surface by sliding (also called “growth-powered passive surface translocation”), which is driven by the outward pressure of cell growth. This process, which does not require flagella (Martínez et al., 1999), occurs due to surfactants, which decrease the surface tension and allow the spread of cells from their origin (Kearns, 2010). Several studies have indicated that lipooligosaccharides (LOSs), glycolpeptidolipids (GPLs), phthiocerol dimycocerosates (PDIMs), and phenolic glycolipids (PGLs) on the outer surface of mycobacteria have important functions in sliding motility (Ren et al., 2007; Tatham et al., 2012; Pang et al., 2013; Mohandas et al., 2016).

Sliding motility allows the colony diameter of non-swarming bacteria to increase during prolonged incubation (Kearns, 2010). Mycobacteria are prototypical non-flagellated microorganisms that spread slowly in a uniform monolayer due to sliding motility (Shi et al., 2011; Maya-Hoyos et al., 2015). Furthermore, sliding movements and biofilm formation facilitate diffusion and colonization by mycobacteria (Pang et al., 2013).

A biofilm is a thin and slimy film of one or more species of bacteria that adhere to each other and/or a solid surface and it can help to increase the virulence of the bacterial species. Biofilm formation may be considered a survival “strategy” for bacteria. There are several consecutive stages of biofilm formation: reversible attachment, irreversible attachment, mature biofilm formation, and dispersion. A biofilm consists of bacteria and matrix material, which includes extracellular polymeric substances, such as polysaccharides, lipids, membrane vesicles, and nucleic acids (Sousa et al., 2015; Toyofuku et al., 2015). Bacteria in biofilms have significantly enhanced resistance to antibiotics and the human immune system (Shi et al., 2011). Biofilm formation is highly related to sliding motility in *Mycobacterium* spp. (Nessar et al., 2011; Mohandas et al., 2016). Previous studies indicated that *Mycobacterium smegmatis* with defects in biofilm formation also have impaired sliding motility (Recht and Kolter, 2001). In addition, Ghosh et al. (2013) reported that the ability of *M. smegmatis* to form biofilms declined as sliding motility declined. Many other species of mycobacteria, including *Mycobacterium avium* and *Mycobacterium fortuitum*, are well-known to produce biofilms, although the ability of *M. marinum* to form biofilms remains largely unknown (Ren et al., 2007).

There has been an increased number of cases of *M. marinum i*nfection due to the increasing popularity of home aquariums. However, there is little knowledge about the pathogenic mechanism of this species in humans. Moreover, sliding motility is highly associated with biofilm formation in mycobacteria. In this study, we aimed to identify genes that have roles in sliding motility in *M. marinum*.

## Materials and Methods

### Bacteria Strains

*M. marinum* NTUH-M6885 is a strain that was clinically isolated at the National Taiwan University Hospital. It was cultured in 7H9 medium supplemented with 10% oleic acid/albumin/dextrose/catalase (OADC), 0.5% glycerol, and 0.05% Tween-80 at 32°C (Tan et al., 2006; Chen et al., 2015). *Escherichia coli* DH10B was grown in Luria broth (LB). When required for the experiments, the antibiotic hygromycin was used at a concentration of 50 mg/L for *M. marinum* and 100 mg/L for *E. coli*.

### Generation of *M. marinum* Transposon Mutant Library

*M. smegmatis* mc^2^155 was used to propagate the TM4-derived conditionally replicating phage phAE94 [which was a kind gift from Dr. William R. Jacobs, Jr., Howard Hughes Medical Institute, New York, NY, United States (Bardarov et al., 1997)]. This phage carries the kanamycin-resistance transposon Tn5367 (Shin et al., 2006). We followed the procedures used in previous studies (Rybniker et al., 2003; Chen et al., 2015) to promote phAE94 infection of *M. marinum* NTUH-M6885 cells.

### Screening for *M. marinum* Transposon Mutants With Decreased Sliding Motility

Sliding motility mutations were screened for using a sliding agar plate (7H9, 6% glycerol and 0.3% agarose) (Mohandas et al., 2016). An aliquot of 1 μL bacteria culture (in the stationary phase) was dropped onto these agar plates (24- or 6-wells) and cultured at 32°C for 9–11 days. To confirm the sliding defect in transposon and deletion mutants, 1 μL bacteria culture adjusted to an optical density at 600 nm (OD_600_) of 1 was dropped onto 6-well plates and cultured at 32°C for 7 days. The diameters of the sliding areas were then measured.

### Identification of Transposon Mutants by Semi-Random Polymerase Chain Reaction (PCR)

The insertion sites of Tn5367 in the transposon mutants were identified by semi-random PCR and DNA sequencing as previously described (Chun et al., 1997; Choi et al., 2001; Shin et al., 2006; Chen et al., 2015). Supplementary Table 1 shows the primers used in these experiments.

### Construction of Deletion Mutants

Gene-deleted fragments were constructed using primers listed in Supplementary Table 1. The Hygr-lacZ-sacB cassette of the pGOAL19 plasmid (Addgene plasmid #20190, Cambridge, MA, United States) was digested with ScaI and the gene-deleted fragments were then cloned into the ScaI site of the digested plasmid. We then transformed the constructed plasmid into *M. marinum* NTUH-M6885 by electroporation (BTX^®^, ECM 630 Electroporation System, MA, United States) under the following conditions: 2500 mV, 1,000 Ω, and 25 μF (Larsen et al., 2007). Following the procedure of Parish and Stoker (2000) and Chen et al. (2015), *M. marinum* unmarked deletion mutants (with in-frame deletions) were obtained after two rounds of homologous recombination. After recovering from the electroporation, each transformed *M. marinum* mutant was cultured on 7H11 with 50 mg/L hygromycin for 2 weeks. The single colony was then subcultured on 7H11 with 100 mg/L X-gal and 50 mg/L hygromycin for 1 week to select the single cross-over transformants (blue colonies). The blue colonies were selected and cultured on 7H11 with 2% sucrose and 100 mg/L X-gal for 1 week to obtain the deletion mutants (white colonies).

### 5′-Rapid Amplification of cDNA Ends (RACE)

The 5′-RACE procedure was performed using a SMARTer^TM^ RACE cDNA Amplification Kit^1^ (CA, United States). This kit includes SMARTer II A oligonucleotides and SMARTScribe Reverse Transcriptase. This allowed isolation of the complete 5′ sequence of our target transcript from the total RNA.

### Construction of Complemented Strains

After isolating the complete 5′ sequence of the target operon, we amplified and connected the promoter region and target genes using overlap- or inverse-PCR, with primers listed in Supplementary Table 1. The complementation fragments were then cloned into a blunted HindIII-site of pMN437 (which was a kind gift from Dr. Michael Niederweis, University of Alabama at Birmingham, Birmingham, AL, United States) (Steinhauer et al., 2010). Subsequently, we transformed the complementation plasmid into deletion mutants.

### Growth Curve

Cultures were inoculated with fresh precultures to an OD_600nm_ of 0.1. Bacterial growth was monitored spectrophotometrically, and colony counts were determined every day.

### Biofilm Formation

*M. marinum* was cultured in 7H9 medium (without Tween-80) with OADC, and with shaking at 100 rpm (Pang et al., 2012; Mohandas et al., 2016). The cell concentration was adjusted to an OD_600nm_ of 0.01 in round-bottom 96-well polypropylene cell culture plates (costar^®^ 3879, New York, NY, United States) at 32°C, and the cells were then cultured for 3 weeks. After 3 weeks, the medium was removed. We stained the biofilm with 200 μL 1% crystal violet (CV) for 10 min (Trivedi et al., 2016). We then removed the CV and washed the biofilm twice with 1× phosphate-buffered saline (PBS). Subsequently, we extracted the CV from the biofilm using 99.5% EtOH and assessed the biofilm formation by measuring OD_490nm_.

### Confocal Laser Scanning Microscopy

*M. marinum* was cultured on cover slides with 7H9 medium (without Tween-80) with OADC, and with shaking at 100 rpm for 2 weeks. The bacteria were then washed with PBS before fixing in formalin. The biofilm was observed with a Leica TCS SP5 confocal laser scanning microscope (Leica, Wetzlar, Germany) and three-dimensional images were analyzed using Volocity software (version 6.0.1, MA, United States).

### Western Blotting

Bacteria were cultured in 7H9 medium for 4 days (to mid-log phase). We then removed the culture medium and washed the bacteria with Sauton’s medium. The bacteria were then cultured for 4 days at 32°C (Gao et al., 2004). Culture filtrates collected by centrifugation and filtration through 0.22 μm-pore-size polyethersulfone filter were condensed using a concentrator (Amicon, NJ, United States) with a 3-kDa cut-off. Next, 10 μg proteins were loaded on 15% sodium dodecyl sulfate (SDS)-polyacrylamide gel electrophoresis (PAGE) gel. The proteins were detected using antibodies against 10 kDa culture filtrate protein (CFP-10; which is also known as EsxB), Ag85B (Ag85B protein levels was used for normalization), and heat-shock protein 65 (Hsp-65; also called grOEL; Hsp-65 protein levels was used for normalization). The details regarding the antibodies were are follows: anti-CFP-10 antibody (Abcam, ab45074, 1:5000), anti-Ag85B antibody (Abcam, ab43019, 1:3000), and anti-Hsp-65 antibody (grOEL; Abcam, ab20519, 1:200).

### Two-Dimensional Thin-Layer Chromatography (2D-TLC)

The extraction and 2D-TLC analysis of polar lipids was based on established procedures used in our previous study (Chen et al., 2015) and in a study by Burguiere et al. (2005). First, the polar lipids were extracted from *M. marinum* grown on 7H11 agar plates. Second, lipids were examined using TLC aluminum sheets (Merck, Summit, NJ, United States). Third, the LOS signals were visualized by spraying the plates with ceric ammonium molybdate [CAM; 24 g (NH_4_)_6_M_o7_O_24_⋅4H_2_O, 0.5 g ammonium cerium nitrate, 500 mL H_2_O, and 28 mL H_2_SO_4_], followed by gentle charring of the plates (Chen et al., 2015).

### Statistical Analysis

All the data were from three independent experiments and they are presented as means ± standard deviations (SDs). We estimated the statistical significance of the differences using one-way analysis of variance (ANOVA) or two-tailed Student’s *t*-tests using GraphPad Prism software (version 5.01, La Jolla, CA, United States).

## Results

### Screening an *M. marinum* Transposon Library for Sliding Motility

We first constructed a transposon library of *M. marinum* NTUH-M6885 containing 2,304 mutants to screen for genes associated with sliding motility. We then randomly selected 16 of these mutants and used semi-random PCR and sequencing to characterize them. The results showed that these 16 mutants had unique transposon insertion sites (Supplementary Table 2), indicating that the library had good diversity.

Next, we screened the 2,304 mutants for sliding motility using 24-well sliding agar plates. We then re-examined and identified 13 mutants with defects in sliding motility using 6-well sliding agar plates. The wild-type strain slid to the edges of the culture well (3.5 cm in diameter), whereas the 13 mutants had sliding distances <2 cm (Supplementary Figure 1). Finally, we reperformed the same experiment using an equal quantity of bacteria cultured for 7 days (OD_600_ = 1), and we found that only five mutants had sliding motility defects (Supplementary Figure 2).

### Role of the Early Secreted Antigen 6 kDa (ESAT-6) Secretion System 1 (ESX-1) Genes in Sliding Motility

**FIGURE 1:**
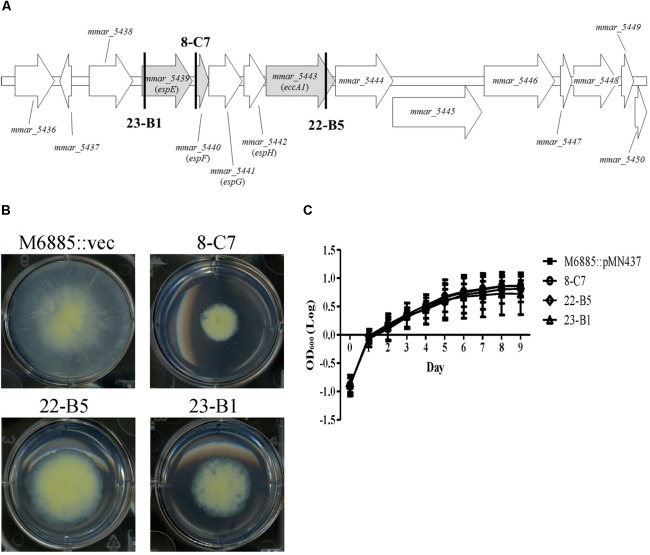
Transposon disruption of three ESX-1-related genes significantly impairs sliding motility but not growth. **(A)** Transposon insertion sites within the ESX-1 locus. The three disrupted genes (gray) were *espE* (*mmar_5439*), *espF* (*mmar_5440*), and *eccA1* (*mmar_5443*). The bold vertical lines indicate the transposon insertion sites. **(B)** Sliding motility of the three transposon mutants (8-C7, 22-B5, and 23-B1). Aliquots of 1 μL bacteria culture (OD_600_ = 1) were added to 6-well sliding agar plates and cultured at 32°C for 1 week. **(C)** Bacteria were cultured in 7H9 medium supplemented with 10% oleic acid/albumin/dextrose/catalase (OADC), 0.5% glycerol, and 0.05% Tween-80. The growth of different strains was assessed based on OD_600nm_. Here and below, all data are from three independent experiments and presented as means ± SDs with one-way ANOVA.

We identified the disrupted genes of the five transposon mutants with sliding motility defects using semi-random PCR and DNA sequencing (**Table 1**). Two mutants harbored insertions within *tetR* and *phoU*, which both belong to putative regulators. The other three transposon mutants—23-B1 (*mmar_5439*), 8-C7 (upstream of *mmar_5440*), and 22-B5 (*mmar_5443*)—had disruptions in the gene cluster encoding the type VII secretion system ESX-1 (**Figures 1A,B**). Previous studies named *mmar_5439* as *espE*, *mmar_5440* as *espF*, and *mmar_5443* as *eccA1* (Bitter et al., 2009; Sani et al., 2010). Among these gene candidates, we focused on the ESX-1 cluster containing *espE*, *espF*, and *eccA1*. The products of all three of these genes are supposed to be displayed at the cell surface but their functions regarding sliding motility have not been characterized in *M. marinum*. These initial results suggested that ESX-1-related genes could be involved in sliding motility.

**Table 1:** Transposon mutants with reduced sliding motility.

Mutant (library no.)	Genes inserted by transposon	Putative function
12-B1	*mmar_4631*	TetR family transcriptional regulator
14-A7	*mmar_4859*	Phosphate transport system regulatory protein PhoU
23-B1	*mmar_5439*	Secretion protein EspE
8-C7	upstream of *mmar_5440*	Secretion protein EspF
22-B5	*mmar_5443*	Type VII secretion AAA-ATPase EccA1


### Detection of Growth Rates in Transposon Mutants With Sliding Defects

The presence of a surfactant in the culture medium and a high bacterial growth rate can increase sliding motility (Kearns, 2010). Thus, it is necessary to determine whether the impaired sliding of the transposon mutants resulted from a growth defect. Recovery of bacterial counts (data not shown) and monitoring changes in OD_600nm_ over time was carried out to measure the growth rates of the wild-type strain and its three transposon mutants (23-B1, 8-C7, and 22-B5). Based on assays in general culture medium (7H9 with 10% OADC, 0.5% glycerol, and 0.05% Tween-80) or sliding broth medium (7H9 with 0.5% glycerol), the growth rates of the three transposon mutants showed no significant differences compared to the corresponding growth of the wild-type strain (**Figure 1C** and Supplementary Figure 3A). Thus, the sliding defects of these three mutants were not due to defects in growth.

### Transcriptional Units and Sequence Alignments of the ESX-1 Gene Cluster

**FIGURE 2:**
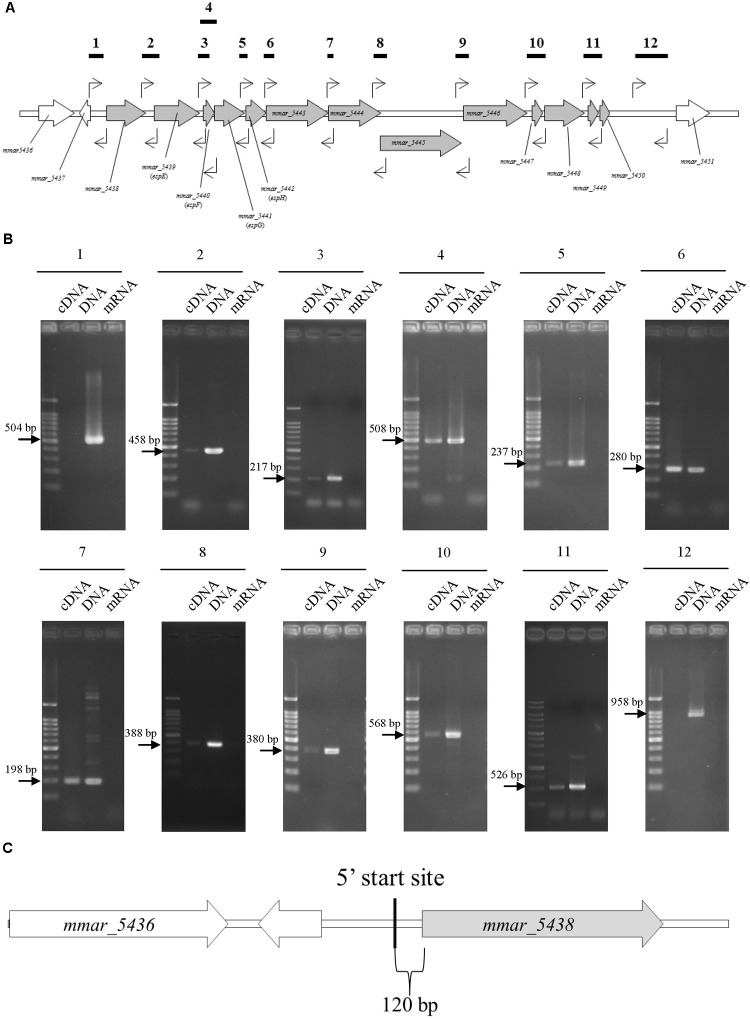
The sliding-related operon involves *mmar_5438* to *mmar_5450*, and its 5′start site is 120 bp upstream of *mmar_5438*. The primer pairs were: 1, 5437-38 gap-F/5437-38 gap-R; 2, 5438-39 gap-F/5438-39 gap-R; 3, 5439-40 gap-F/5439-40 gap-R; 4, 5439-40 gap-F/5440-41 gap R; 5, 5441-42 gap-F/5441-42 gap-R; 6, 5442-43 gap-F/5442-43 gap-R; 7, 5443-44 gap-F/5443-44 gap-R; 8, 5444-45 gap-F/5444-45 gap-R; 9, 5445-46 gap-F/5445-46 gap-R; 10, 5446-47 gap-F/5447-48 gap-R; 11, 5448-49 gap-F/5449-50 gap-R; 12, 5450-51 gap-F/5450-51 gap-R2. The primer list is shown in Supplementary Table 1. **(A)** Primer recognition sites. The bold horizontal lines indicate amplified gene fragments, and arrows indicate primers. **(B)** Data showing that the sliding-related operon involves *mmar_5438* to *mmar_5450*. Each gel is independent and not cropped from different parts of the same gel. cDNA: cDNA of *M. marinum* NTUH-M6885 (template); DNA: DNA of *M. marinum* NTUH-M6885 (positive control); mRNA: mRNA of *M. marinum* NTUH-M6885 (negative control, to exclude genomic DNA contamination). **(C)** The 5′ start site begins 120 bp upstream of *mmar_5438* (gray). The bold vertical line indicates the 5′ start site, detected using a SMARTer^TM^ RACE cDNA Amplification Kit.

Reverse-transcription (RT)-PCR to determine the transcriptional units of the ESX-1 gene cluster was performed with total RNA from *M. marinum* NTUH-M6885 as the template and primer pairs that hybridize within two consecutive genes. Total RNA without RT was used as the negative control, to exclude genomic DNA contamination. **Figures 2A,B** showed positive results for the junctions of genes from *mmar_5438* to *mmar_5450*. In addition, Supplementary Figures 4A,B show the longer amplified PCR products containing two or three adjacent genes. The PCR products with expected sizes were also confirmed by sequencing. These results indicate that the ESX-1 gene cluster contains 13 genes that belong to a single transcriptional unit. This operon starts with *mmar_5438* and ends with *mmar_5450*.

Next, we identified the 5′ start site of this operon using a SMARTer^TM^ RACE cDNA Amplification Kit. The result showed that the transcriptional start site of the ESX-1 operon was located 120 bp upstream of *mmar_5438* (**Figure 2C**). Furthermore, we determined the full DNA sequence of this operon in *M. marinum* NTUH-M6885 using next-generation sequencing. Analysis of the nucleotide sequences of the ESX-1 cluster from *M. marinum* NTUH-M6885 (National Center for Biotechnology Information [NCBI] accession number: MF034931) revealed 99% sequence similarity compared with the cluster from the *M. marinum* M strain.

### Roles of *espE*, *espF*, *espG*, and *espH* Genes in Sliding Motility

**FIGURE 3:**
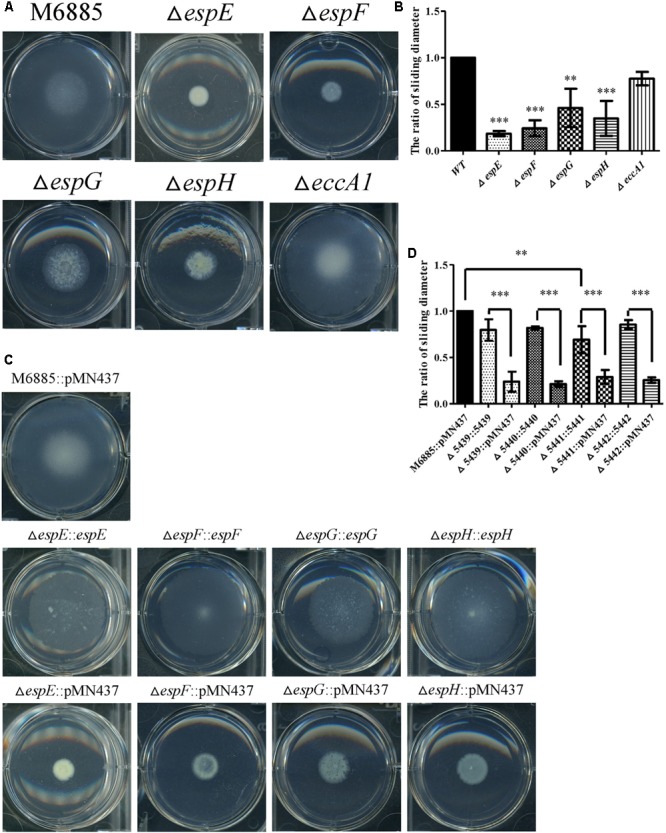
Four mutants (*espE*, *espF*, *espG*, and *espH*) have significantly impaired sliding motility. **(A)** Sliding motility of *M. marinum* deletion mutants (∆*espE*, ∆*espF*, ∆*espG*, ∆*espH*, and ∆*eccA1*) and *M. marinum* NTUH-M6885 (wild-type) on sliding agar plates. **(B)** Quantification of sliding diameters of the 6 strains shown in **(A)**. **(C)** Sliding motility of complemented strains relative to *M. marinum* NTUH-M6885 harboring pMN437 on sliding plates. **(D)** Quantification of sliding diameters of the nine strains in **(C)**. The quantification of the sliding diameters was used for normalization, with the wild-type diameter being assigned a value of 1. Means and SDs from three independent experiments were calculated with one-way ANOVA (^∗∗^*p* < 0.01, ^∗∗∗^*p* < 0.001).

The genes, *espE* (*mmar_5439*), *espF* (*mmar_5440*), and *eccA1* (*mmar_5443*) are all located in the same operon. To further characterize whether the specific genes of the ESX-1 cluster play roles in sliding motility, unmarked deletion mutants of *espE*, *espF*, and *eccA1* and also of *espG* (*mmar_5441*) and *espH* (*mmar_5442*), were constructed. The deletions of *espE*, *espF*, *espG*, *espH*, and *eccA1* were validated by PCR using two primer pairs targeted to the deleted genes and their flanking regions (Supplementary Figure 5). **Figures 3A,B** show that the sliding motility of each unmarked deletion mutant, except for the *eccA1* mutant (∆*eccA1*), was dramatically reduced compared with the sliding motility of the wild-type strain. The motility defects of the gene deletion mutants were significantly restored by complementation with the corresponding gene (**Figures 3C,D**). The growth rates of these deletion mutants were not significantly different compared to the growth rate of the wild-type strain (Supplementary Figure 3B). However, the sliding motility of the *espG*-complemented strain was only partially restored by complementation with the plasmid containing the *espG* gene. As the ESX-1 operon contains the promoter for 13 genes, the results imply that proper regulation of *espG* gene expression might require *cis*-elements within the operon. Thus, these results suggest that *espE*, *espF*, *espG*, and *espH* have important roles in sliding motility in *M. marinum* NTUH-M6885.

### Roles of *espE*, *espF*, *espG*, and *espH* Genes in Biofilm Formation

**FIGURE 4:**
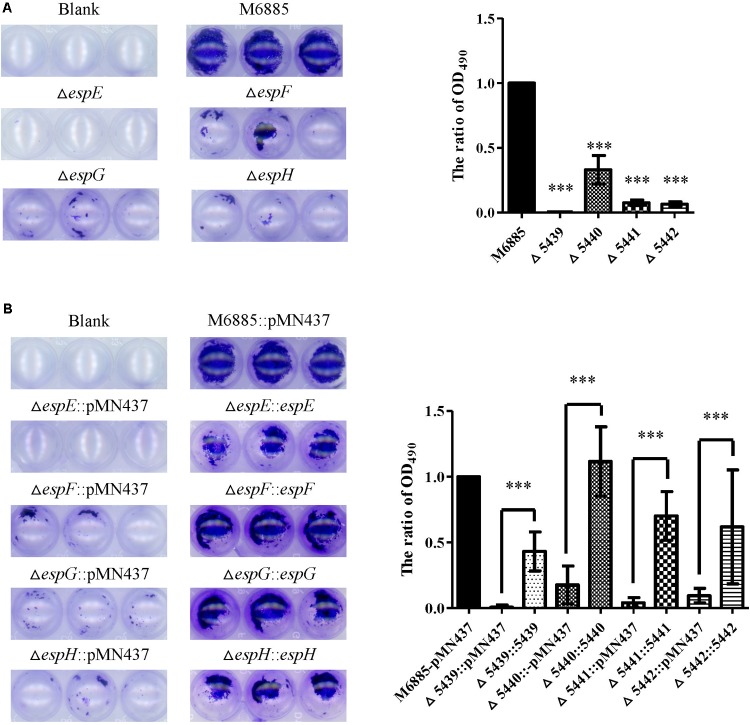
The *espE*, *espF*, *espG*, and *espH* genes also function in biofilm formation. Cells were stained with 1% CV and OD_490nm_ was measured after 10 min. **(A)** Biofilm formation by the deletion mutants (∆*espE*, ∆*espF*, ∆*espG*, and ∆*espH*) and *M. marinum* NTUH-M6885 (wild-type), and quantitation of these results in all four strains (right). The quantification of biofilm was used for normalization, with the wild-type diameter being assigned a value of 1. All deletion mutants were compared with the wild-type strain. Means and SDs from three independent experiments from triplicates were calculated with one-way ANOVA (^∗∗∗^*p* < 0.001). **(B)** Biofilm formation in complemented strains, and quantification of these results in all 9 strains (right). The quantification of biofilm was used for normalization, with the wild-type diameter being assigned a value of 1. All deletion mutants were compared with the wild-type strain and their corresponding complemented strain. Means and SDs from three independent experiments from triplicates were calculated with one-way ANOVA (^∗∗∗^*p* < 0.001).

**FIGURE 5:**
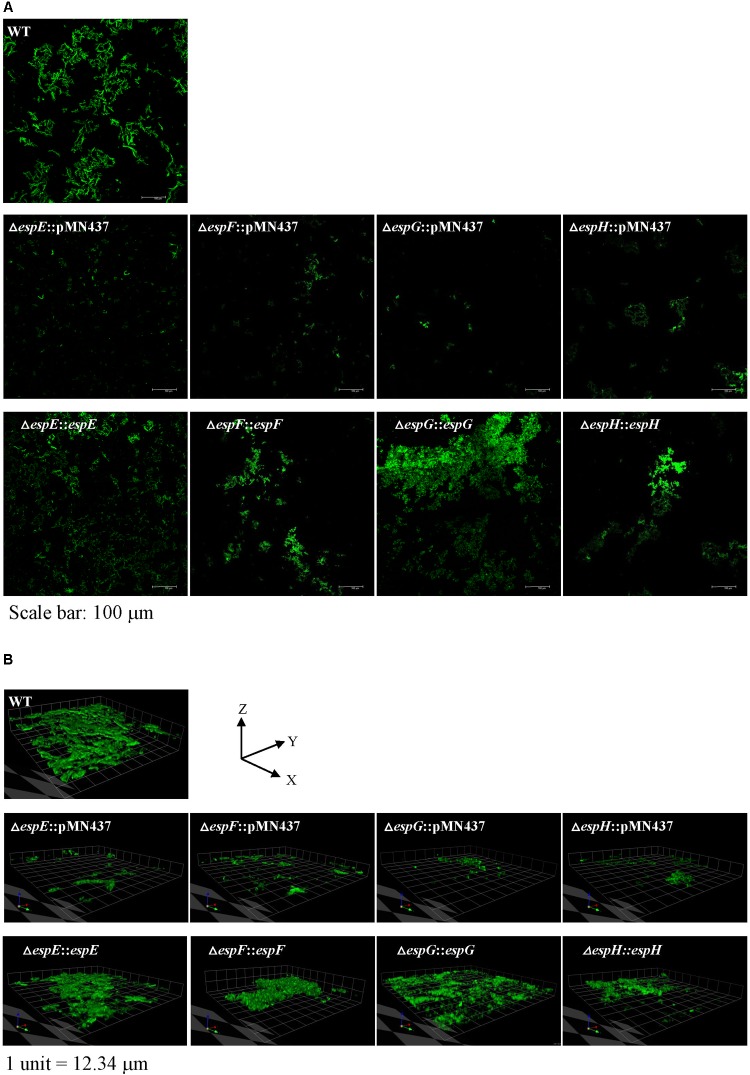
Visualization of biofilm formation on cover slides with confocal laser scanning microscopy (CLSM). To further analyze the structure of the biofilms, CLSM was performed using bacteria carrying pMN437, which harbors *gfp*. **(A)** Microscopic image at 20× magnification. Scale bar = 100 μm. **(B)** 3D imaging with a Z-stack of biofilm structure.1 unit = 12.34 μm.

Previous studies indicated that sliding motility correlates with biofilm formation in *Mycobacterium* spp. (Nessar et al., 2011; Sousa et al., 2015; Mohandas et al., 2016). To discern whether the ESX-1-related genes contribute to biofilm formation, we compared the biofilm formation of the deletion mutants with that of the wild-type strain. After 3 weeks of culturing in general culture medium without Tween-80, the deletion mutants exhibited decreased biofilm formation compared with the wild-type strain. All complemented strains produced biofilms similar to that of the wild-type strain (**Figure 4**). To further confirm the role of these ESX-1 genes in biofilm formation, three-dimensional biofilms were formed on cover slides and they were analyzed using confocal laser scanning microscopy (**Figure 5**). The biofilms of these deletion mutants were thinner and more scattered than those of the wild-type strain, and complementation significantly restored biofilm formation. These data indicate that *espE*, *espF*, *espG*, and *espH* are required for biofilm formation in *M. marinum*.

### EsxB Secretion in Δ *espE*, Δ *espF*, Δ *espG*, and Δ *espH* Mutants

According to previous studies, both EsxA (ESAT-6) and EsxB (CFP-10) are indispensable components of the ESX-1 secretion system (Ates et al., 2016; Wong, 2017) and they are considered to be indicators of the ESX-1 system with secretion function (Gao et al., 2004; Brodin et al., 2006; Champion et al., 2014). To investigate whether *M. marinum* NTUH-M6885 had a functional ESX-1 system, EsxB secretion from the wild-type strain was detected by western blotting. Cell filtrate of *M. tuberculosis* H37Rv was used as a positive control (Supplementary Figure 6). The *M. marinum* NTUH-M6885 wild-type strain and also all of the Esx-1 gene deletion mutants accumulated EsxB protein in their cell lysates. However, deletion of *espE*, *espF*, *espG*, and *espH* led to 0.35-, 0.59-, 0.82-, and 0.73-fold lower secretion of EsxB protein than the secretion of the wild-type strain. These results indicate that wild-type *M. marinum* NTUH-M6885 could secrete EsxB protein. There was a substantial decrease in secretion for *espG* and *espH* deletion mutants, and a slight decrease for *espE* and *espF* deletion mutants.

## Discussion

The *M. marinum* NTUH-M6885 transposon mutant library was screened, and 5 mutants (8-C7, 12-B1, 14-A7, 22-B5, and 23-B1) that had defective sliding motility were identified. 12-B1 and 14-A7 had an interruption in *tetR* and *phoU*, respectively. Both genes are putative regulators and they might regulate the other downstream effector genes for sliding motility directly or indirectly. Further studies are needed to understand whether the regulation of *tetR* or *phoU* is critical for sliding motility in *M. marinum*. 22-B5 had a transposon insertion in the *eccA1* gene and showed significantly decreased sliding motility; however, the sliding motility of the *eccA1* gene deletion strain was similar to that of the wild-type strain. Several studies have reported that the insertion of transposable elements influences the expression of nearby genes (Wei and Cao, 2016) (which is known as the polar effect). The expression levels of genes both upstream and downstream of insertion sites can be affected (Cheng et al., 2015). Quantification of mRNA indicated that the expression of neighboring genes (*espE*, *espF*, and *espG*) was reduced in strain 22-B5 (Supplementary Figure 7). Thus, the sliding defect in strain 22-B5 was due to a polar effect. The virulence of the *eccA1* transposon mutants in previous studies could be fully complemented (Gao et al., 2004; Joshi et al., 2012), indicating that *eccA1* affects virulence through a mechanism unrelated to the effect on sliding ability. The discrepancy might be due to the virulence of *M. marinum* not being caused by sliding ability.

A previous study showed that EccA1 plays a regulatory role in mycolic acid synthesis in *M. marinum* (Joshi et al., 2012). In addition, alterations in mycolic acid synthesis and composition can affect biofilm formation in *M. smegmatis* (Ojha et al., 2005). In the present study, the deletion of *eccA1* in *M. marinum* did not significantly affect sliding motility and biofilm formation (data not shown), indicating that alterations in the mycolic acid synthesis in the *eccA1* mutant do not correlate with sliding motility and biofilm formation in *M. marinum*.

The *mmar_5438*-*mmar_5450* gene cluster of *M. marinum* and the *Rv3863-Rv3875* gene cluster of *M. tuberculosis* share 79% identity based on nucleotide sequence analysis. Bottai et al. (2011) previously revealed the operon structures of the ESX-1 gene cluster in *M. tuberculosis*, showing that *espE*, *espF*-*espG*, and *espH*-*eccA1* belong to three independent operons. However, our results demonstrated that the operon structure of the ESX-1 loci in *M. marinum* involves a single operon. Therefore, we speculate that the ESX-1 gene cluster in different mycobacteria species might have different sequence alignments and operon structures.

Previous research indicated that EspE and EspF are major cell surface proteins in *M. marinum* (Sani et al., 2010; van der Woude et al., 2012). EspG proteins are cytosolic chaperones that specifically interact with heterodimeric PE-PPE substrates (Ates et al., 2016) and EspH is required for substrate export (EsxA and EsxB) (Gao et al., 2004; McLaughlin et al., 2007; Champion et al., 2014). In addition, a previous study showed that a defect in *M. marinum espE* led to the formation of smooth colonies (Carlsson et al., 2009). In agreement with this finding, we also found morphological changes in ∆*espE*, ∆*espF*, ∆*espG*, and ∆*espH* mutants and smooth colonies were observed (data not shown). Several studies have reported that morphological change correlates with reduced sliding motility (Maya-Hoyos et al., 2015; Lee et al., 2017). These might be the reasons why the ∆*espE* and ∆*espF* mutants had more severe defects in sliding motility and biofilm formation.

*espE* encodes the major surface-associated protein in *M. marinum* (Sani et al., 2010). The deletion of *espE* resulted in the most profoundly reduced sliding motility and biofilm formation abilities. To further investigate whether the surface properties of the *espE* mutant were changed, the surface charge and hydrophobicity of the wild-type and ∆*espE* strains were determined by using a Zetasizer Nano ZS (Malvern, Malvern, United Kingdom) (Kundu et al., 2017) and a bacterial adhesion to solvent assay (Miyamoto et al., 2004; Xu et al., 2009). Our results showed that the surface charge and hydrophobicity of the *espE* mutant were not significantly different compared with the wild-type values (data not shown). The LOS biosynthesis of ∆*espE* was assessed by 2D-TLC and was similar to that of the wild-type strain (Supplementary Figure 8). Thus, we suppose that the decreased sliding ability and biofilm formation of the *espE* mutant were not due to the alterations of the surface charge, hydrophobicity, or LOS biosynthesis.

Our results indicate that the deletion of *espE*, *espF*, *espG*, and *espH* resulted in extremely decreased sliding motility and biofilm formation. The incomplete restoration exhibited by complemented strains might be due to different expression levels of complemented genes carried by the plasmids, which was reported previously (Karnholz et al., 2006; Lin et al., 2006). *M. marinum* NTUH-M6885 could secrete EsxB protein and had a functional ESX-1 system. In addition, our data show that *espE*, *espF*, *espG*, and *espH* mutants accumulated EsxB protein in the cytosol and had reduced EsxB protein in the cell filtrates when compared with the wild-type strain (Supplementary Figure 6B). These results are consistent with those of a previous study that indicated that an *M. marinum espE* transposon mutant could not secrete EsxB protein and accumulated it in the cytosol (Carlsson et al., 2009). EsxB plays roles in cell membrane lysis and virulence (Unnikrishnan et al., 2017). Several previous studies showed that the secretion of the EsxA-EsxB heterodimer is co-dependent on the secretion of the EspA-EspC heterodimer, suggesting that the EspA-EspC heterodimer has an important function in ESX-1 secretion (Abdallah et al., 2007; Ates and Brosch, 2017; Wong, 2017). Additionally, the signal motifs of EspE and EspF were similar to those of EspA and EspC based on an analysis using the structural homology server PHYRE2 (Solomonson et al., 2015). Sliding motility and biofilm formation correlate with virulence in *Mycobacterium* spp. (Schorey and Sweet, 2008; Pang et al., 2013). Esp proteins regulate substrate export, which might be involved in sliding motility and biofilm formation.

The ESX-1 system is one of the major groups of T7SS (Bottai et al., 2011; Korotkova et al., 2014) and its role in pathogenesis has been reported in *M. tuberculosis* (Unnikrishnan et al., 2017; Wong, 2017) and *M. marinum* (Gao et al., 2004; Cardenal-Munoz et al., 2017; Unnikrishnan et al., 2017). Previous studies reported that *espG* and *espH* affect *M. marinum* virulence by influencing cytolysis, cytotoxicity, growth in macrophages, and spreading (Gao et al., 2004; McLaughlin et al., 2007). Bottai et al. (2011) indicated that *espF* and *espG1* were associated with virulence during *M. tuberculosis* infection (Brodin et al., 2006) and *M. tuberculosis* can survive in bone marrow-derived macrophages. The present study is the first study to demonstrate the contribution of the ESX-1 system to sliding motility and biofilm formation in *M. marinum*.

In summary, we demonstrated that *espE*, *espF*, *espG*, and *espH* genes are critical for sliding motility and biofilm formation in *M. marinum*. These genes, which are located in the T7SS ESX-1 operon, are important virulence factors in *M. marinum*.

## Author Contributions

T-LL and J-TW designed the research, discussed the analysis, and revised the paper. L-YL and Y-YC prepared materials and performed experiments. L-YL, Y-YC, T-LL, and P-FH analyzed the data. L-YL, T-LL, and P-FH wrote the main text of the manuscript. All the authors reviewed and approved the final version of the manuscript.

## Conflict of Interest Statement

The authors declare that the research was conducted in the absence of any commercial or financial relationships that could be construed as a potential conflict of interest.

## Footnotes

1 www.clontech.com
