# Supplementary material for: Role of the Mycobacterium marinum ESX-1 Secretion System in Sliding Motility and Biofilm Formation
Source: Front Microbiol. 2018 May 30;9:1160. doi: 10.3389/fmicb.2018.01160 (PMC5988883; doi:10.3389/fmicb.2018.01160)
Supplement: Supplementary file 1 [file Data_Sheet_1.pdf]

Role of the *Mycobacterium marinum* ESX-1 Secretion System in Sliding Motility and Biofilm Formation

Li-Yin Lai[1], Tzu-Lung Lin[1], Yi-Yin Chen[3], Pei-Fang Hsieh[1], Jin-Town Wang*[1,2]

[1]Department of Microbiology, National Taiwan University College of Medicine, Taipei, Taiwan.

[2]Department of Internal Medicine, National Taiwan University Hospital, Taipei, Taiwan.

[3]Department of Pediatrics, Chang Gung Children's Hospital, Chang Gung Memorial Hospital, Chang Gung University, College of Medicine, Taoyuan, Taiwan.

*** Correspondence:**

Jin-Town Wang, M.D., Ph.D.

Dept. of Microbiology

National Taiwan University College of Medicine

1, Sec 1, Jen-Ai Rd.

Taipei, Taiwan

wangjt@ntu.edu.tw

Supplementary Table 1. Primers and plasmids used in this work

Name of primer	Sequence	Purpose	Reference
TnF	TGCAGCAACGCCAGGTCCACACT	Semi-random PCR	Chen et al., 2015
TnR	CAGAAAGTCGTCAGGTCAGC	Semi-random PCR	Chen et al., 2015
HOPS1	GGCGTAGGAACCTCCATCATC	Semi-random PCR	Bardarov et al., 1997
HOPS2	CTTGCTCTTCCGCTTCTTCTCC	Semi-random PCR	Bardarov et al., 1997
semi-rand_2-1	GGCCACGCGTCGACTAGTACNNNNNNNNNNGCAGC	Semi-random PCR	Choi et al., 2001
semi-rand_4	GGCCACGCGTCGACTAGTAC	Semi-random PCR	Choi et al., 2001
MMAR_RS27300-qF	GGG AAC GCG GCT GAA GT	mmar_5436 mRNA level	
MMAR_RS27300-qR	GCG AAG TGT CCG GTA ATC GT	mmar_5436 mRNA level	
MMAR_5437-qF	GGA AAC CGT TGC GCT CTG	mmar_5437 mRNA level	
MMAR_5437-qR	GGG TGT TGT GAT TCC GGA AG	mmar_5437 mRNA level	
MMAR_5438-qF	GAG GCA AGG CGT TGA CGT T	mmar_5438 mRNA level<br>*espE* deletion<br>Operon analysis	
MMAR_5438-qR	TGT CTT CTT CAG GCG AAT TCG	mmar_5438 mRNA level	
MMAR_5439-qF	TCC CAA GGC GGT CAA CAG	*espE* mRNA level<br>PCR check *espF*<br>Operon analysis	
MMAR_5439-qR	GCA CCG TCT TCG TCG TCT TC	*espE* mRNA level<br>PCR check *espE*<br>Operon analysis	
MMAR_5440-qF	TGA ACG TCG TGC CTT CAT TC	*espF* mRNA level<br>PCR check *espF*	
MMAR_5440-qR	GTT GGT CGC CGA TTT GAG TT	*espF* mRNA level<br>PCR check *espE*<br>Operon analysis	

MMAR_5441-qF	ATG TCG TCG GAG TCG AGG TAA	*espG* mRNA level<br>PCR check *espG*
MMAR_5441-qR	CAG GGA AAT CGA CCA GGT GTA A	*espG* mRNA level<br>PCR check *espF*<br>PCR check *espG*<br>Operon analysis
MMAR_5442-qF	CAC GGG CTG ACC GAG AAG	*espH* mRNA level<br>PCR check *espH*<br>PCR check *eccA1*
MMAR_5442-qR	GGG TTG GTC ACG GTG AAC AT	*espH* mRNA level<br>PCR check *espH*
MMAR_5443-qF	GAC ACC TAC AGC CCG GAA GA	*eccA1* mRNA level<br>PCR check *eccA1*<br>Operon analysis
MMAR_5443-qR	GTC ATT CCC AGC CGC AAT	*eccA1* mRNA level
MMAR_5443-qR2	CTT GCA GTC TGT CCA CAT CG	PCR check *eccA1*
MMAR_5444-qF	GCG ATG CTG GCG GAC TAT	*mmar_5444* mRNA level
MMAR_5444-qR	GAT CGA GTG GCG TCC CTT T	*mmar_5444* mRNA level<br>PCR check *eccA1*<br>Operon analysis
MMAR_5445-qF	CGT TGT GGT GGA CGA GTT TG	*mmar_5445* mRNA level
MMAR_5445-qR	GGT CGA ACA GGC CGA TGA	*mmar_5445* mRNA level
5437-38 gap F	CGT ACA GAG CAG TTG CAG TC	Operon analysis
5437-38 gap R	GCC GAG CAG TCG ATT CGG	Operon analysis
5438-39 gap F	TCG GAA ACC GCG CAA GCC	Operon analysis<br>PCR check *espE*
5438-39 gap R	TTG AAG ATA TCG CCC GGG TC	Operon analysis
5439-40 gap F	AGG GCA AGG CAA CGA GGG	Operon analysis
5439-40 gap R	GTC GCC GAT TTG AGT TCG C	Operon analysis
5440-41 gap F	GCG AAC TCA AAT CGG CGA C	PCR check *espH*
5440-41 gap R	ACA TCG TCA CCG GTG CCC	Operon analysis

		espE deletion
5441-42 gap F	AGC TTC CTC CCC GGT ACC	Operon analysis
5441-42 gap R	AGT CCA GAG TCC TCA GAC G	Operon analysis PCR check *espF*
5442-43 gap F	TTC GCC ACC CGC TAC GAG	Operon analysis
5442-43 gap R	TCG AGA TCT GCG CCG TGC	Operon analysis
5443-44 gap F	AGG CAA TCG CCA CGG TGC	Operon analysis
5443-44 gap R	CGA CAA CGA TGC CCA GCG	Operon analysis PCR check *espH*
5444-45 gap F	GTA CTA CAT CGA CCC GGA AG	Operon analysis
5444-45 gap R	TGA TCA CGA TCA TGC CCA GC	Operon analysis
5445-46 gap F	GCA CGC TCT ATA TCA GTG GG	Operon analysis
5445-46 gap R	TCG TCC ATG ATT CCC AGC G	Operon analysis
5446-47 gap F	CCC TTA CAT CGA GCC GCC	Operon analysis
5447-48 gap R	CGG TAT TCA GCT CCG GTG G	Operon analysis
5448-49 gap F	GAC GAG GCC GAC GAA GAC	Operon analysis
5449-50 gap R	TGC GCT GGA TGC TGC CTC	Operon analysis
5450-51 gap F	CGG AGG CTG CTG CAC AAC	Operon analysis
5450-51 gap-R2	TGC AGC CAC GCG AAG GAC	Operon analysis
5439-inverse-F-in	TCC GGG CAA CCG GCG G	*espE* deletion
5439-inverse-R-in	TCG CGG AAT TCC TTA CAC TCG	*espE* deletion
MMAR5440-Ff-F	AAAGGCCTCGAAAAGGTGCC	*espF* deletion Operon analysis
MMAR5440-Ff-R	AGCGGACCGGTCATAGGGGGTAGACCTTTC	*espF* deletion
MMAR5440-Rf-F	GAAAGGTCTACCCCCTATGACCGGTCCGCT	*espF* deletion
MMAR5440-Rf-R	GCGTACTCGTCGAGTGCACC	*espF* deletion
MMAR5441-Ff-F	CACCCAAGATCGACATTCCG	*espG* deletion
MMAR5441-Ff-R	ACGGAGAGGATTAAGATCAGCCAAAAATCT	*espG* deletion

MMAR5441-Rf-F	AGATTTTTGGCTGATCTTAAT CCTCTCCGT	*espG* deletion
MMAR5441-Rf-F	ACACCAGATGCTCCGAACCG	*espG* deletion
MMAR5442-Ff-F	AGGCCTGCAGGGCGTCACG G	*espH* deletion
MMAR5442-Ff-R	GCGATCAGTCATGTATGTGG CGTCCCTTTC	*espH* deletion
MMAR5442-Rf-F	GAAAGGGACGCCACATACAT GACTGATCGC	*espH* deletion
MMAR5442-Rf-R	TGCTTGCTGGGCTGTGCGAC	*espH* deletion
MMAR5443-Ff-F	AACCCGCCAATGTCGAACCG	*eccA1* deletion Operon analysis
MMAR5443-Ff-R	GCGAAGCCCCATGTTGTATC ACCGTTCGTT	*eccA1* deletion
MMAR5443-Rf-F	AACGAACGGTGATACAACAT GGGGCTTCGC	*eccA1* deletion
MMAR5443-Rf-R	TTGAGGGTCTCGTCGGGCAG	*eccA1* deletion
5439-complement-R	CTA GAG GAG GGT CCC CTC	*espE* complementation
Inverse 39 F	ATG GTG CCA AAG GGA AGC GG	*espE* complementation PCR check *espE*
Inverse 39 R	GTA CGT CGA TTC CTC GCT CG	*espE* complementation
5` start site (complementation) F	GTT CCA CTG CGC AAT GCT TC	*espE, F, G* and *H* complementation
5`start-R2-5440	GTC CTG TCA TGT ACG TCG ATT CCT CGC TCG	*espF* complementation
5` start site (complementation) R-5441	GAG CGG ACC GGT CAT GTA CGT CGA TTC CTC	*espG* complementation
5` start site (complementation) R-5442	CCC GGG CAG GTC CAC GTA CGT CGA TTC CTC	*espH* complementation
mmar_5440 F (complementation)	GAG GAA TCG ACG TAC ATG ACA GGA CTA CTG	*espF* complementation
mmar_5440 R (complementation)	TCA GCC AAA AAT CTT GTC GA	*espF* complementation PCR check *espF*
mmar_5441 F (complementation)	GAG GAA TCG ACG TAC ATG ACC GGT CCG CTC	*espG* complementation

mmar_5441 R (complementation)	TCA ACC TCG GGC GGT GG	*espG* complementation
mmar_5442 F (complementation)	GAG GAA TCG ACG TAC GTG GAC CTG CCC GGG	*espH* complementation
mmar_5442 R (complementation)	TCA CCG TTC GTT GTA ACG AG	*espH* complementation
inverse 39 F	ATGGTGCCAAAGGGAAGCGG	Operon analysis
5444 F	CTGGCAATTGCCACCCATC	Operon analysis
5445 F	TCACGAACTCAAGACCGGC	Operon analysis
5446 F	CCGACATGGAAGTGGCTTAC	Operon analysis
5448 F	ATGACGTCGATCCTCGATCC	Operon analysis

Supplementary Table 2. Diversity of transposon mutants

Library no.	Genes disrupted by transposon	Insertion sequence
6-A5	Between *mmar_5417* and *mmar_5418*	tgaaactt
6-A8	Between *mmar_3099* and *mmar_3100*	cctttagc
6-A9	*mmar_3070*	acaaatgg
6-A10	*mmar_1029*	tctaatgt
6-A11	Between *mmar_3415* and *mmar_3416*	caaatta
6-A12	Between *mmar_2822* and *mmar_2823*	gcaatagc
6-B5	*mmar_5392*	cctaactc
6-B9	*mmar_3548*	acatttcc
6-B12	*mmar_1361*	ccatttgg
6-C5	No similarity with sequences of *M. marinum*	tctttagg
6-C7	*mmar_2318*	gtaaaggg
6-C12	*mmar_3182*	gaatttgg
6-D2	*mmar_0932*	tagtttgg
6-D4	*mmar_2766*	acatttga
6-D6	No similarity with sequences of *M. marinum*	tcaattgc
6-D7	No similarity with sequences of *M. marinum*	gctataca

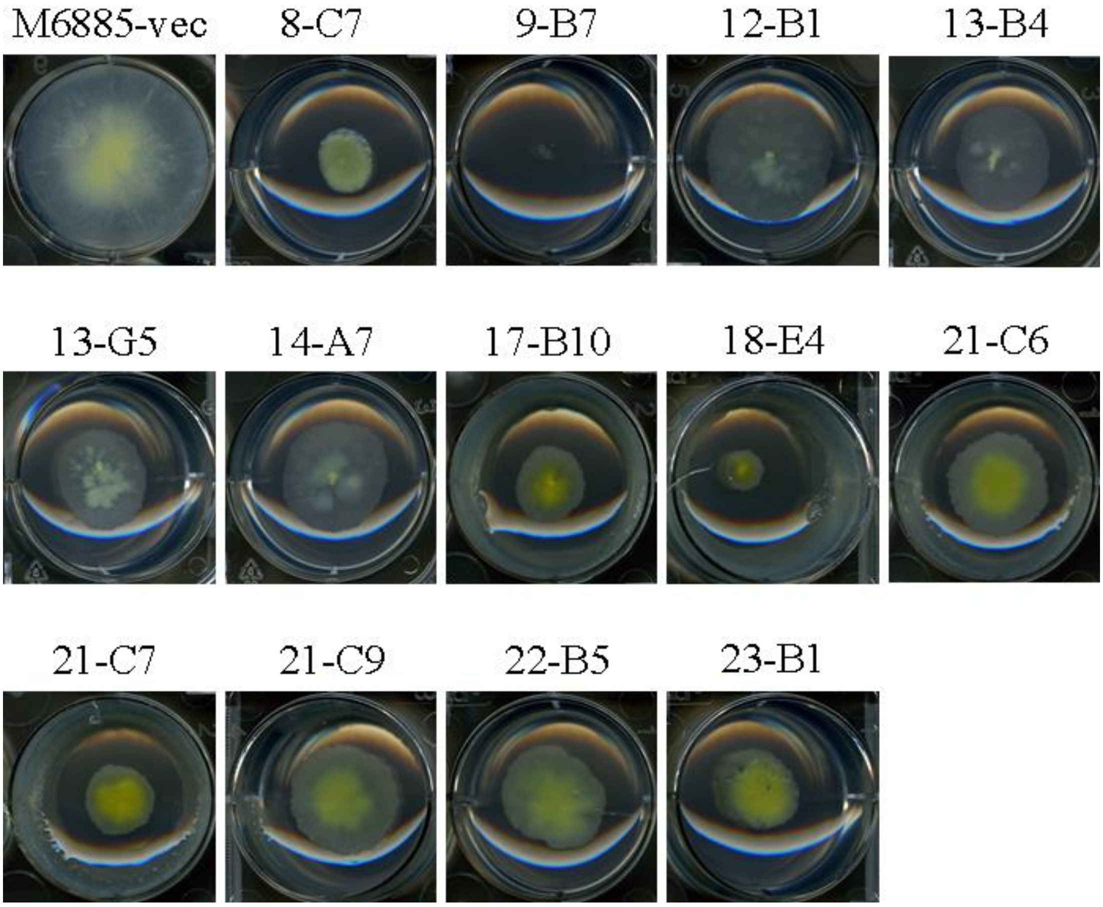

Supplementary Figure 1. Sliding motility in transposon mutants during stationary phase culture.

Sliding motility of transposon mutants was determined by adding a 1 μL drop from a stationary phase culture onto 6-well sliding plates.

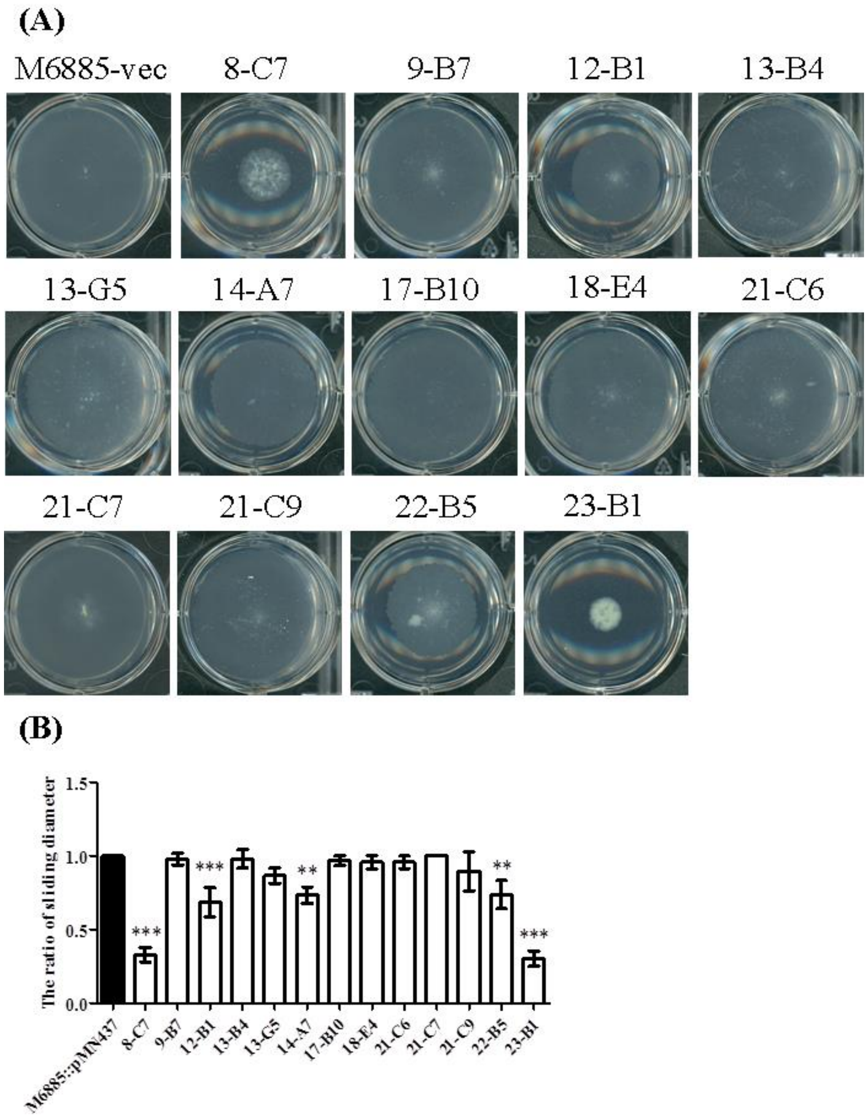

Supplementary Figure 2. Sliding motility in transposon mutants when the cultures had an OD_{600nm} of 1.

(A) A 1 μL drop from a bacterial culture ($OD_{600} = 1$) was placed onto 6-well sliding plates. **(B)** Quantification of the results in (A). All data were from three independent experiments and presented as means ± SDs with one-way ANOVA (**$p < 0.01$, ***$p < 0.001$). The quantification of sliding diameters was used for normalization, with the wild-type diameter being assigned a value of 1.

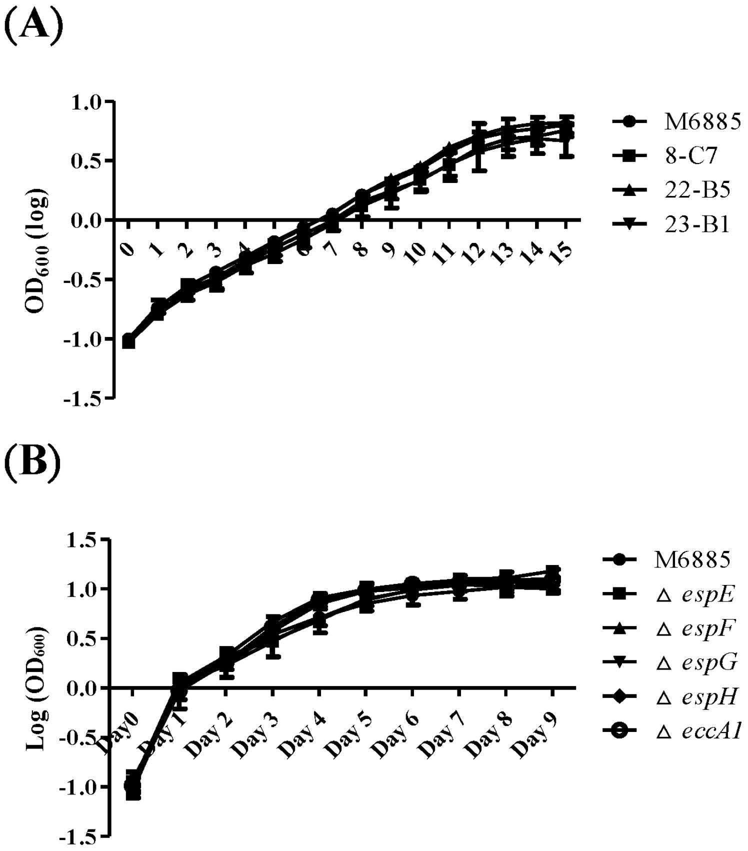

Supplementary Figure 3. Growth curves of transposon mutants, deletion mutants (△*espE*, △*espF*, △*espG*, △*espH* and △*eccA1*), and the wild-type strain. **(A)** Growth curves of transposon mutants and the wild-type strain. OD_{600nm} was measured for 15 days for cultures in sliding broth. **(B)** Growth curves of deletion mutants and the wild-type strain. OD_{600nm} was measured for 9 days in 7H9 medium with OADC and Tween-80. All data were from three independent experiments and presented as means ± SDs with one-way ANOVA.

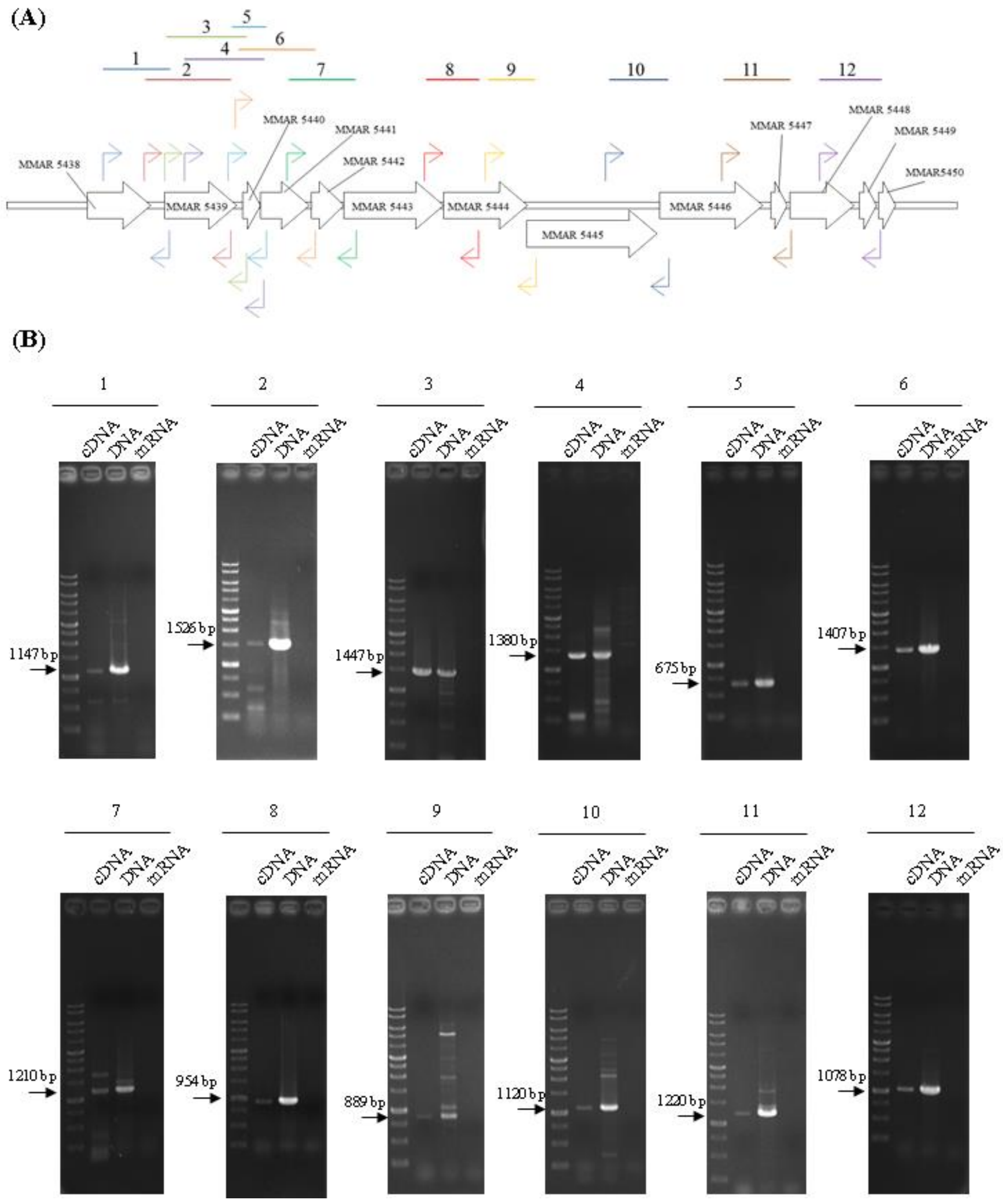

Supplementary Figure 4. The sliding-related operon involves *mmar_5438* to *mmar_5450*.

The primer pairs were: 1, MMAR_5438-qF/ 5438-39 gap R; 2, 5438-39 gap F/ MMAR_5439-qR; 3, inverse 39 F/ MMAR_5440-qR; 4, MMAR5440-Ff-F/ 5440-41 gap R; 5, MMAR_5439-qF/ MMAR_5441-qR; 6, 5439-40 gap F/ 5441-42 gap R; 7,

MMAR5443-Ff-F/ 5442-43 gap R; 8, MMAR_5443-qF/ MMAR_5444-qR; 9, 5444 F/ 5444-45 gap R; 10, 5445 F/ 5445-46 gap R; 11, 5446 F/ 5447-48 gap R; 12, 5448 F/ 5449-50 gap R. The primer list is in Supplementary Table 1. **(A)** Primer recognition sites. The bold horizontal lines indicate amplified gene fragments, and arrows indicate primers. **(B)** Data showing that the sliding-related operon involves *mmar_5438* to *mmar_5450*. Each gel is independent and not cropped from different parts of the same gel. cDNA: cDNA of *M. marinum* NTUH-M6885 (template); DNA: DNA of *M. marinum* NTUH-M6885 (positive control); mRNA: mRNA of *M. marinum* NTUH-M6885 (negative control, to exclude genomic DNA contamination).

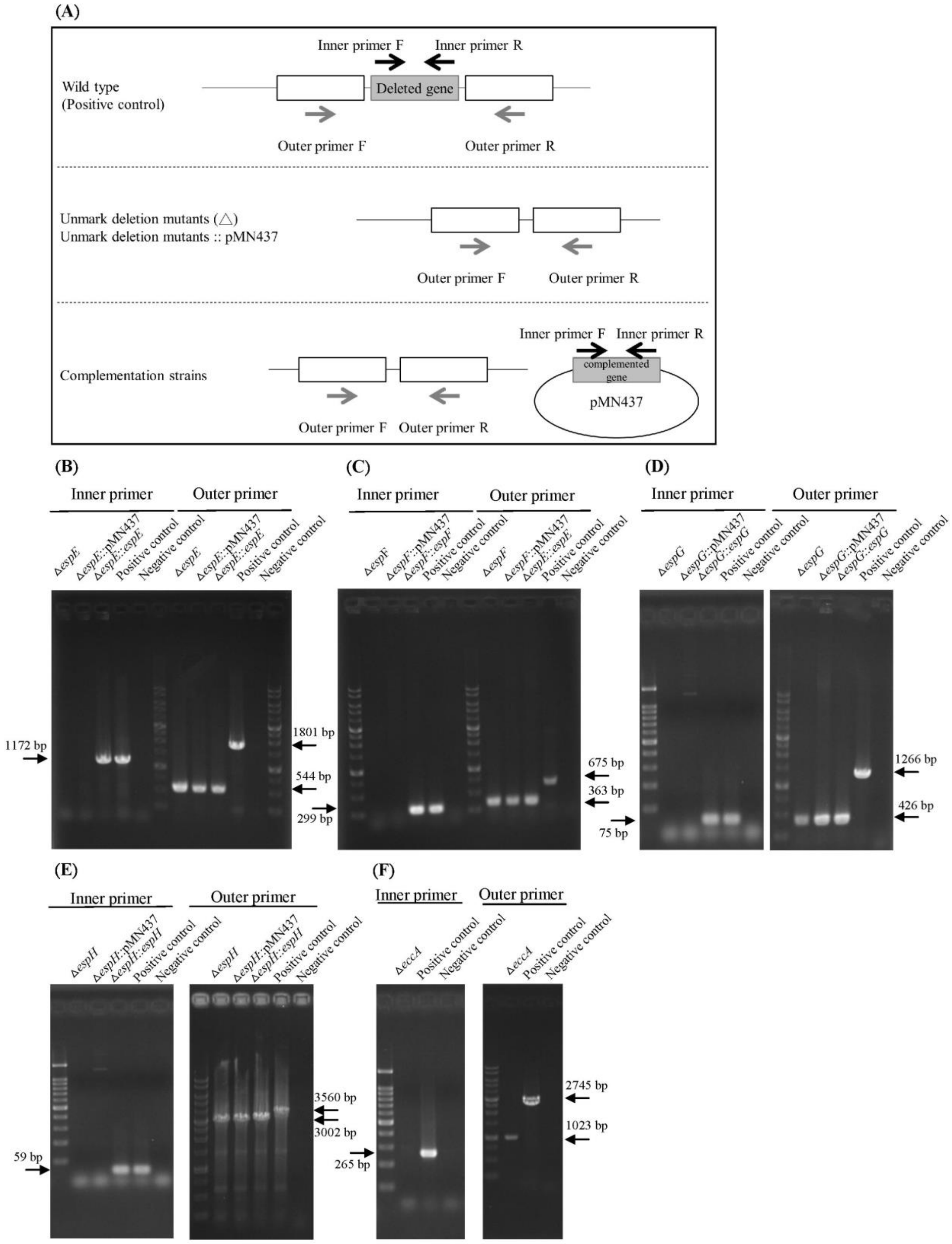

Supplementary Figure 5. PCR verification of the genotype of deletion mutants (△*espE*, △*espF*, △*espG*, △*espH* and △*eccA1*), complementation strains, and the wild-type strain.

frontiers

(A) Illustration of PCR reaction sites in different constructed strains. Black arrow: inner primer pairs targeted to the deleted genes. Gray arrow: outer primer pairs targeted to the flanking regions. **(B)** Inner primer pairs for *espE*: inverse 39-F and MMAR_5439-qR; outer primer pairs for *espE*: 5438-39 gapF and MMAR_5440-qR. **(C)** Inner primer pairs for *espF*: MMAR_5440-qF and mmar_5440 R (complementation); outer primer pairs for *espF*: MMAR_5439-qF and MMAR_5441-qR. **(D)** Inner primer pairs for *espG*: MMAR_5441-qF and MMAR_5441-qR; outer primer pairs for *espG*: MMAR_5440-qF and 5441-42 gap R. **(E)** Inner primer pairs for *espH*: MMAR_5442-qF and MMAR_5442-qR; outer primer pairs for *espH*: 5440-41 gap F and 5443-44 gap R. **(F)** Inner primer pairs for *eccA1*: MMAR_5443-qF and MMAR_5443-qR2; outer primer pairs for *eccA1*: MMAR_5442-qF and MMAR_5444-qR. The positive control was wild-type DNA. The negative control was H_2O. All primer sequences are shown in Supplementary Table 1.

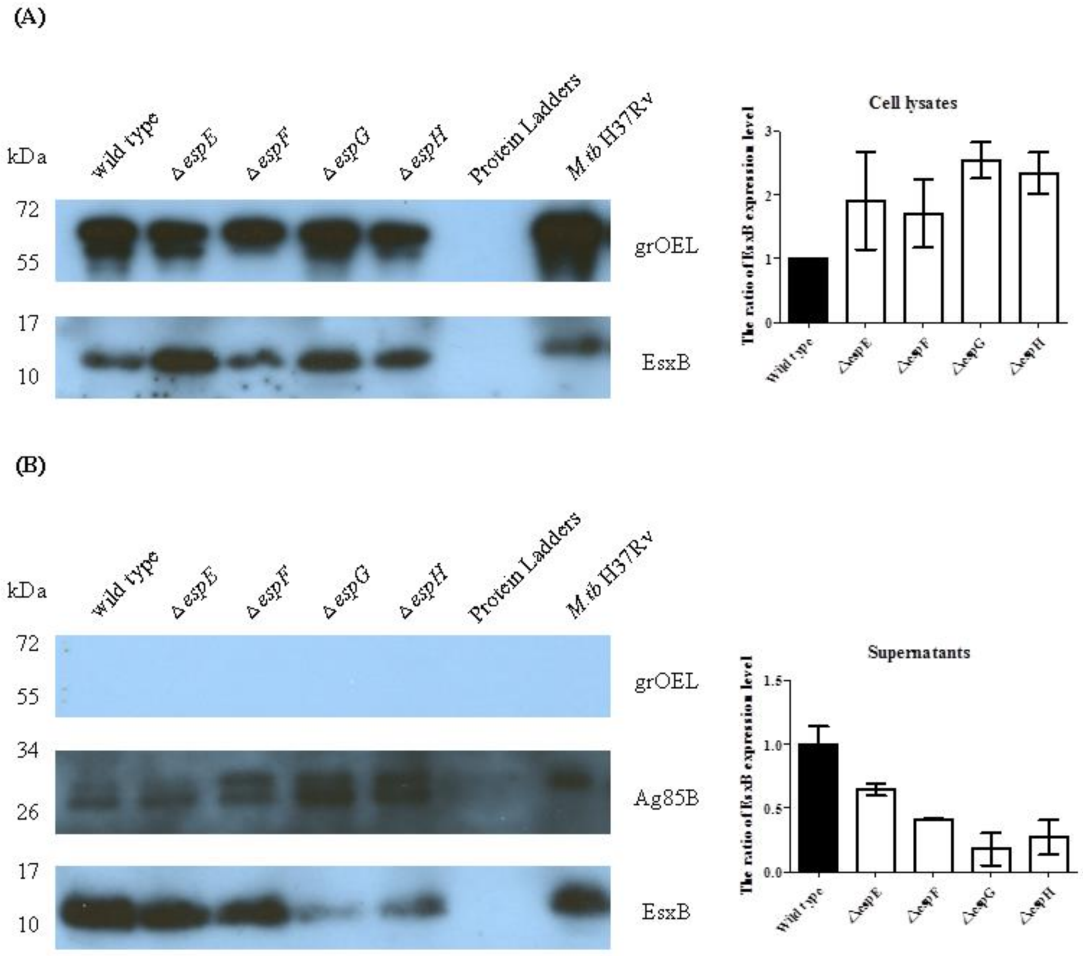

Supplementary Figure 6. Western blot assay of the EsxB secretion in deletion mutants (Δ*espE*, Δ*espF*, Δ*espG*, and Δ*espH*).

(A) Cell lysates of the wild-type strain and deletion mutants were analyzed by western blot (left). Densitometry analysis of the western blots (right). The quantification of grOEL protein levels was used for normalization, with the wild-type diameter being assigned a value of 1. **(B)** Supernatants of the wild-type strain and deletion mutants were analyzed by western blot (left). Densitometry analysis of the western blots (right). The quantification of Ag85B protein levels was used for normalization, with the wild-type diameter being assigned a value of 1. All the strains had ESX-1 secretion ability. The Δ*espG* and Δ*espH* mutants had slightly reduced EsxB secretion. The cell fraction of *M. tuberculosis* H37Rv was used as a positive control. 20 μg of cell lysates was separated by 15% SDS-PAGE. 10 μg of cell filtrations was separated by 15% SDS-PAGE. Densitometry analysis was based on two independent experiments.

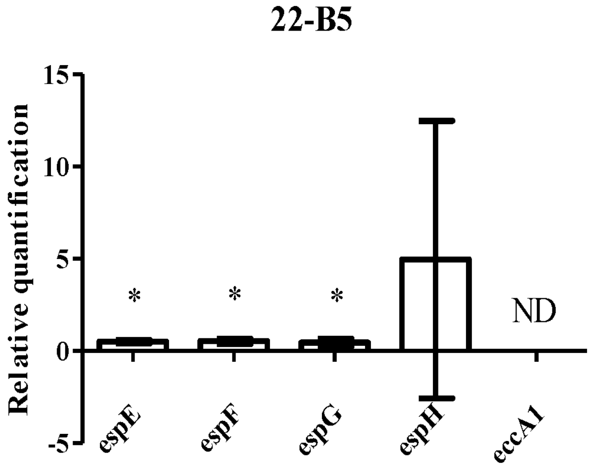

Supplementary Figure 7. RT-PCR to detect the *espE*, *espF*, *espG*, and *espH* gene expression levels in 22-B5.

The quantification of mRNA expression levels was normalized based on the wild-type strain. The results indicated that the gene expression levels of *espE*, *espF*, and *espG* decreased significantly in 22-B5. All data are from three independent experiments and they are presented as means ± SDs with unpaired two-tailed Student's *t*-tests. ND: not determined. *: $P < 0.05$.

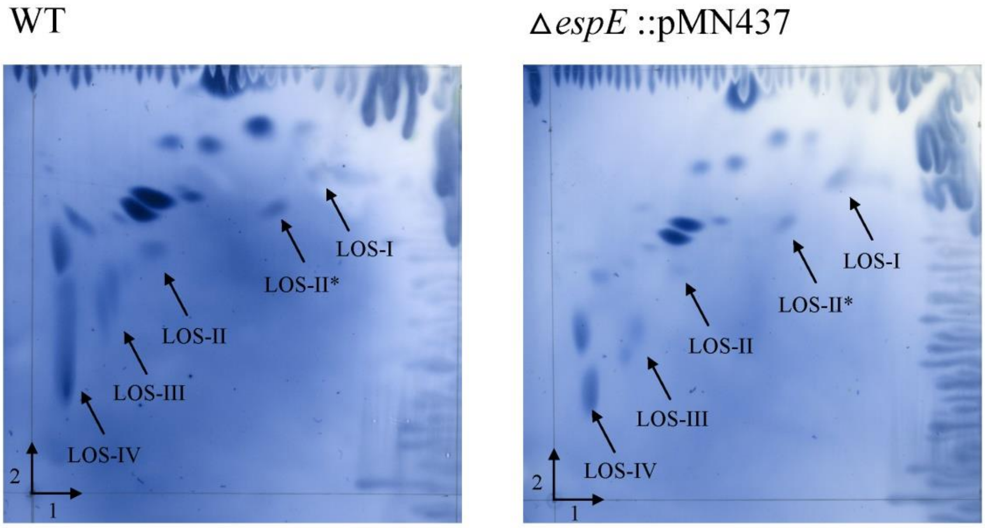

Supplementary Figure 8. Detection of lipooligosaccharide (LOS) biosynthesis in the wild-type strain and Δ*espE* mutant.
Two-dimensional thin- layer chromatography (2D-TLC) was used to analyze the polar lipids in *M. marinum*. The polar lipids were separated by chloroform/methanol/water (20:10:2, v/v/v) in the first direction and by chloroform/acetic acid/methanol/water (20:12.5:1.5:2, v/v/v/v) in the second direction. The plates were sprayed with ceric ammonium molybdate (CAM) to visualize the LOS signals.
